# Identification of key genes and pathways associated with different immune statuses of hepatitis B virus infection

**DOI:** 10.1111/jcmm.14616

**Published:** 2019-09-29

**Authors:** Jinglan Jin, Hongqin Xu, Ruihong Wu, Na Gao, Na Wu, Shibo Li, Junqi Niu

**Affiliations:** ^1^ Department of Hepatology, The First Hospital of Jilin University Jilin University Changchun China; ^2^ Jilin Province Key Laboratory of Infectious Diseases Laboratory of Molecular Virology Changchun China; ^3^ Department of Infectious Disease The Third Affiliated Hospital of Sun Yat-sen University Guangzhou China; ^4^ Lanshan People’s Hospital Linyi China; ^5^ Department of Pediatrics, Genetics Laboratory University of Oklahoma Health Sciences Center (OUHSC) Oklahoma City OK USA

**Keywords:** DNA methylation profile, Gene expression profile, IgG Fc receptors, T cell receptor signalling pathway, TLR signalling pathway

## Abstract

We aimed to identify key genes and pathways associated with different immune statuses of hepatitis B virus (HBV) infection. The gene expression and DNA methylation profiles were analysed in different immune statuses of HBV infection. Differentially expressed genes (DEGs) and differentially methylated genes (DMGs) were identified, followed by their functional and integrative analyses. The differential expression of IgG Fc receptors (FcγRs) in chronic HBV‐infected patients and immune cells during different stages of HBV infection was investigated. Toll‐like receptor (TLR) signalling pathway (including TLR6) and leucocyte transendothelial migration pathway (including integrin subunit beta 1) were enriched during acute infection. Key DEGs, such as FcγR Ib and FcγR Ia, and interferon‐alpha inducible protein 27 showed correlation with alanine aminotransferase levels, and they were differentially expressed between acute and immune‐tolerant phases and between immune‐tolerant and immune‐clearance phases. The integrative analysis of DNA methylation profile showed that lowly methylated and highly expressed genes, including cytotoxic T lymphocyte‐associated protein 4 and mitogen‐activated protein kinase 3 were enriched in T cell receptor signalling pathway during acute infection. Highly methylated and lowly expressed genes, such as Ras association domain family member 1 and cyclin‐dependent kinase inhibitor 2A were identified in chronic infection. Furthermore, differentially expressed FcγR Ia, FcγR IIa and FcγR IIb, CD3^−^CD56^+^CD16^+^ natural killer cells and CD14^high^CD16^+^ monocytes were identified between immune‐tolerant and immune‐clearance phases by experimental validation. The above genes and pathways may be used to distinguish different immune statuses of HBV infection.

**Highlights**

1. Innate immune responses mediated by TLR signalling may regulate acute HBV infection.
2. Leucocyte transendothelial migration pathway was involved in HBV immune clearance.
3. FcγR Ia, FcγR IIa and FcγR IIb may serve as potential targets for HBV infection.
4. *CTLA4* and *MAPK3* genes may be involved in acute HBV infection.
5. Methylation of *RASSF1A* and *CDKN2A* genes may be involved in chronic HBV infection.

## 1 INTRODUCTION

Hepatitis B virus (HBV), an common human pathogen, spreads through the mucosal and percutaneous exposure to infected blood and other body fluids.^1^ Acute and chronic infection, cirrhosis and hepatocellular carcinoma (HCC) are common sequelae of HBV infection that represents a major health problem worldwide.^2^, ^3^, ^4^ An approximate estimate of 600 000 HBV‐related deaths are annually reported,^5^ and 73% deaths related to liver cancer are attributed to hepatitis virus infection.^6^ Moreover, HCC is known as one of the most common causes of cancer‐related death worldwide.^7^, ^8^ Therefore, a better understanding of molecular mechanisms underlying the progression of HBV infections may facilitate researchers to design specific biomarkers and effective therapeutic strategies for HBV‐related liver diseases.

In general, the co‐ordinate action of both innate and adaptive immune responses is believed to be involved in the sustained control of HBV infection.^9^, ^10^ Innate immunity is the early defensive line for viral containment and may efficiently induce virus‐specific adaptive responses through the production of pro‐inflammatory cytokines and chemokines.^11^ Several innate effectors are found to exhibit adaptive‐like features or exert defensive effects against HBV via immunoregulation of T cells.^12^ Moreover, the frequency of natural killer (NK) cells is found to increase during the early stages of HBV infection and decreases with the decrease in viraemia,^13^ indicative of the key roles of innate immune responses following HBV exposure. Adaptive immunity necessitates time to activate the functional maturation and expansion of distinct B and T cell clones, which can specifically recognize the infectious agent and generate a memory response to enhance the host ability to control the infection caused by the same pathogen.^14^ HBV infection outcome is ultimately determined by the presence of functional HBV‐specific T cells and antibody‐producing B cells.^15^ Despite great efforts, the immunopathogenesis of HBV infection is largely unknown.

Accumulating studies have identified key molecules that play a crucial role in regulating the immune response against HBV infections. HBV X protein has been shown to disrupt innate immunity through the down‐regulation of mitochondrial antiviral signalling protein.^16^ Toll‐like receptors (TLRs) may activate intracellular antiviral pathways and promote the production of antiviral effectors such as interferons (IFNs) to further activate immune responses for controlling HBV infection.^17^ In addition, aberrant cytosine‐guanine dinucleotide (CpG) DNA methylation is shown to be correlated with the progression of liver diseases caused by chronic HBV infection.^18^ A study has confirmed that HBV infection progression is associated with the methylation rate of exportin 4 (XPO4) promoter and that XPO4 methylation status may be used as a promising biomarker to predict HBV infection progression.^19^ However, the molecular mechanisms that activate the immune responses to prevent HBV infections are questionable.

To identify key genes and pathways associated with the different immune statuses of HBV infection, integrative analysis of gene expression and DNA methylation profiles of HBV infection were performed in this study. Moreover, the differential expression of IgG Fc receptors (FcγRs) in chronic HBV‐infected patients and immune cells at different stages of chronic HBV infection was investigated. The findings of this study will aid in the development of a promising biomarker to predict HBV infection progression.

## 2 MATERIALS AND METHODS

### 2.1 Workflow of this study

**Figure 1:**
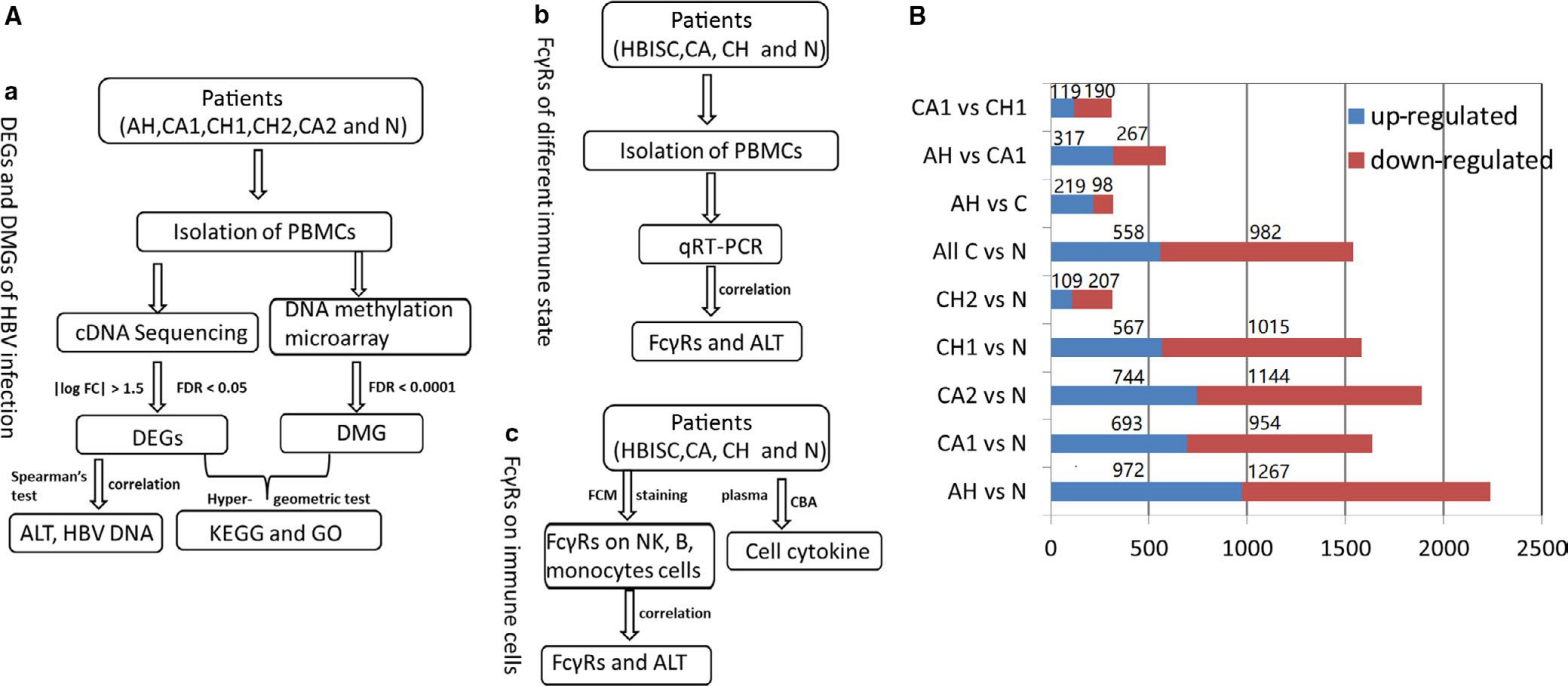
The workflow of this study (A) and different expressed genes between different immune state of HBV infection patients (B). (a) Integrative analysis of gene expression and DNA methylation profiles of HBV infection; (b) Analysis of FcγRs expression in different immune state of chronic HBV‐infected patients; (c) Differential expression of FcγRs on immune cells in different statuses of HBV infection

This study mainly includes three parts (Figure 1A): (a) identification of key genes and pathways related to different immune statuses of hepatitis B virus by integrative analysis of gene expression and DNA methylation profiles of HBV infection, (b) analysis of the expression of FcγRs in different immune statuses of HBV‐infected patients by qRT‐PCR and (c) expressions of FcγRs and cell cytokines on NK cells, B cells and monocytes in different phases of HVB infection. In addition, the correlation between key genes, FcγRs and clinical indicators were also performed.

#### 2.1.1 Integrative analysis of gene expression and DNA methylation profiles of HBV infection

##### Subjects

A total of 76 HBV patients hospitalized in the First Bethune Hospital of Jilin University and eight healthy individuals who attended physical examination, between December 2008 and December 2010, were recruited. All individuals provided informed consent for using their samples for research. According to the different immune stage and natural history, patients with HBV infection could be divided into acute hepatitis B (AH) and chronic hepatitis B. According to the 2009 update of the American Association for the Study of Liver Diseases (AASLD)^20^ and guidelines for the prevention and control of European liver disease (EASL),^21^ chronic hepatitis B were subdivided into immune‐tolerant (CA1), inactive (CA2), immune‐clearance (hepatitis B e antigen (HBeAg) positive) (CH1) and immune‐clearance (hepatitis B e antigen (HBeAg) negative) (CH2) phases. In accordance with the diagnostic criteria as previously described,^22^ the characteristic AH, CA1, CH1, CH2 and CA2 were listed in Table 1a. Besides, the inclusion criteria for enrolling patients and healthy individuals were also described in detail in Table 1a.

**Table 1:** The diagnostic and inclusion criteria for enrolling HBV‐infected patients and healthy individuals

	Groups/number	Diagnostic criteria	inclusion criteria
a (84)	AH/9	Acute hepatitis B; no history of hepatitis B; negative for the presence of five serological markers of hepatitis B for nearly 2 months; acute onset; HBV surface antigen (HBsAg) and hepatitis B core antibody (HBcAb) IgM positive; and symptoms such as jaundice and pain over the liver area, but no signs of chronic liver disease and cirrhosis, portal hypertension, hypersplenism, and ascites	The inclusion criteria for enrolling patients with HBV infection were as follows: HBsAg positive; ultrasound testing showed no other lesions such as liver cirrhosis and liver cancer; patients had no long history of drinking; patients never received any drugs (including liver‐protective drugs) for long term within half a year; patients never received anti‐HBV therapy, including IFN and nucleoside analogues; patients had no other organ diseases such as hypertension, diabetes and cancer
	CA1/16	Chronic hepatitis B; positive for HBsAg and serum HBV DNA (serum HBV DNA > 2000 IU/mL) for more than 6 mo; HBeAg positive; normal range of alanine aminotransferase (ALT) and aspartate transaminase (AST) for more than consecutive three times within a year; and no abnormality during liver histological examination	
	CH1 (HBeAg+)/30	Chronic hepatitis B; positive for HBsAg and HBeAg for more than 6 months; serum HBV DNA positive (serum HBV DNA > 20 000 IU/mL); persistent or recurrent elevated transaminase or hepatitis lesions during liver histological examination; and no other cause of liver damage	
	CH2 (HBeAg‐)/9	Chronic hepatitis B; positive for HBsAg for more than 6 months; serum HBV DNA positivity (serum HBV DNA 2000‐20 000 IU/mL); negative for HBeAg and/or positive for anti‐HBeAg for more than 6 months; persistent or recurrent elevated transaminase or hepatitis lesions during liver histological examination; and no other cause of liver damage	
	CA2/12	Chronic hepatitis B; positive for HBsAg; serum HBV DNA < 2000 IU/mL; HBeAg negative; normal range of ALT for more than consecutive three times within a year	
	Normal/8	Age ranging from 18 to 65 years; no history of hepatitis B; negative result for the five serological markers of hepatitis B within 1 week; negative results for hepatitis A, C and E antibodies; HIV antibody negativity; no autoantibodies; no obvious abnormality in liver ultrasound; no long history of drinking; no long‐term use of any drugs (including liver‐protective drugs) within half a year; and no other organ diseases such as hypertension, diabetes and cancer	
b (64)	HBISC/12	Acute onset; HBsAg and HBcAb positive; symptoms such as jaundice and uncomfortable liver area, but no signs of chronic liver disease, cirrhosis, and other complications; follow‐up results were HBsAg negative, and no detectable HBV was found	Patients with HBV infection were HBsAg and HBeAg positive among five serological markers of hepatitis B, and other inclusion criteria for enrolling patients with HBV infection were in accordance with the description in Section (a)
	CH/32	HBeAg positive; serum HBsAg and HBVDNA positive for more than 6 months; persistent or recurrent elevated transaminase or hepatitis lesions during liver histological examination; and no other cause of liver damage	
	CA/20	HBeAg positive; serum HBsAg and HBVDNA positive for more than 6 months; normal range of ALT and AST for more than consecutive three times within a year	
	Normal/14	The inclusion criteria for enrolling healthy individuals were in accordance with the description in Section (a)	
c (54)	HBISC/7	The diagnostic and inclusion criteria for enrolling HBV‐infected patients and healthy individuals were in accordance with the description in Section (b)	
	CH/31		
	CA/16		
	Normal/20		

##### Isolation of peripheral blood mononuclear cells (PBMCs)

Whole blood (15 mL) was collected from each patient through peripheral venepuncture and then maintained in the sterile anticoagulant tubes containing sodium citrate (Becton, Dickinson and Company, Franklin Lakes, NJ). After centrifugation at 1200 g/min for 5 minutes, PBMCs were isolated by Ficoll‐Hypaque density gradient separation, counted and saved in RNALater (Qiagen Inc., Valencia, CA) at −80°C.

##### Gene expression microarray preparation, pre‐processing and functional analysis

Using TRIzol (Invitrogen, Corporation, Carlsbad, CA) following the protocols provided by manufacturers, Total RNA was extracted from PBMCs. RNA integrity was then detected performed with Agilent 2100 Bioanalyzer (Agilent Technologies, Santa Clara CA). Equal amount of total RNA (5 µg) was then prepared as microarray targets performed with Ambion RNA amplification kit (Ambion, Austin, TX, USA) for its reverse transcription into cDNA and a single round of in vitro transcription into cRNA. Human WG‐6 v3.0 whole‐genome expression bead array was performed and scanned with Illumina's BeadLab system Array Scanner‐iScan based on Illumina Human whole‐genome gene expression array v3 BeadChip.

All the raw expression data were pre‐processed, including data signal conversion, quality evaluation, probe filtration and normalization. Based on the platform of Illumina Human whole‐genome gene expression array v3, 48 803 probes corresponding to 43 062 transcripts and 24 928 known genes were obtained. After probe filtration and quality evaluation for raw expression data, one CH1 sample and one CH2 sample were excluded. Finally, 82 samples were used for the generation of gene expression profile, including 17 091 probes corresponding to 11 417 genes. The demographic characteristics and clinical features of patients are shown in Table S1. We performed *t* test^23^ to identify differentially expressed genes (DEGs) between two groups such as AH vs normal controls (N), CA1 vs N, CA2 vs N, CH1 vs N, CH2 vs N, all C (CA1, CA2, CH1 and CH2) vs N, AH vs CA1, AH vs CH1, AH vs all C and CA1 vs CH1. By Benjamini‐Hochberg (BH) method,^24^ significant *P*‐value was adjusted as false discovery rate (FDR). Only, DEGs with |log fold change (FC)| > 1.5 and FDR < 0.05 were considered significant.

Gene Ontology (GO, http://www.geneontology.org)^25^ and Kyoto Encyclopedia of Genes and Genomes (KEGG; available at http://www.genome.ad.jp/kegg/)^26^ are common databases used for the function and pathway annotation of large‐scale genes or proteins. In our study, GO and KEGG pathway enrichment analyses were carried out to better understand the biological significance of DEGs performed with the cumulative hypergeometric test. The calculation formula was as follows:

$$P\;=\;1-\sum_{i=0}^{k-1}\frac{C_{M}^{i}C_{N-M}^{n-i}}{C_{N}^{n}}$$

where, N stands for the total number of genes, M represents the number of DEGs, and n and k stand for the total gene number and DEGs corresponding to a special GO function or KEGG pathway, respectively. Significant *P*‐value was adjusted by BH method, and the adjusted *P*‐value < .05 was considered as the cut‐off value.

##### Analysis of the correlation between clinical indicators and gene expression levels

To identify key genes associated with HBV infection, Spearman’s test was performed for the determination of the correlation between gene expression levels and clinical indicators, including ALT levels and HBV DNA. Significant *P*‐value was adjusted by BH method. The adjusted *P*‐value < .05 and |spearman coefficient| > .45 were defined as the cut‐off value.

##### DNA methylation microarray preparation, pre‐processing and functional analysis

Genomic DNA was isolated from the peripheral blood by the overnight digestion of lysed peripheral lymphocytes with proteinase K and phenol/chloroform extraction. The concentration and quality of genomic DNA were determined with NanoDrop 2000 (Thermo Fisher Scientific) and agarose gel electrophoresis (1% wt/vol), respectively. The sample preparation for DNA methylation microarray was carried out as previously described.^27^ To produce 200 to 1000 bp fragments (keeping CpG islands intact), 2 to 6 μg of high‐quality genomic DNA was digested with *Mse*I (New England Biolabs, Ipswich, MA) and then purified using PCR purification kit (Qiagen). The sample was heat‐denatured to form single strands; the methylated DNA fragments were immunoprecipitated (IP) with 1 μg of monoclonal mouse antibody against 5‐methylcytidine (Eurogentec, Belgium) and subsequently captured with Protein A agarose beads. The DNA‐antibody‐bead mixture was digested with proteinase K and purified with phenol‐chloroform. The enrichment of the methylated IP DNA was completed performed with a whole‐genome amplification kit (WGA2 kit, Sigma, USA). The labelling of IP and input DNA, microarray hybridization and scanning were conducted by NimbleGen Laboratories (Reykjavik, Iceland) as previous described.^28^ Data were extracted from scanned images by NimbleGen MS 200 Microarray scanner (48 slides) and data collection software (NimbleGen Systems, Inc, Madison, WI). The samples were assayed in duplicates.

All the methylation microarray data were generated based on Roche NimbleGen Human DNA Methylation 3x720K CpG Island Plus RefSeq Promoter Array and pre‐processed by NimbleScan V2.6 software (Roche) following NimbleScan software user's guide V2.6, including data signal conversion, quality evaluation, probe filtration and normalization. Differentially methylated genes (DMGs) between two groups were identified with *t* test.^23^ Significant *P*‐value was adjusted as FDR by BH method (FDR = 1.11%). A value of *P* < .0001 and the difference in means between the two groups > 0.4 were set as cut‐off values. GO and KEGG pathway enrichment analysis for DMGs were performed with the cumulative hypergeometric test as described above, and the adjusted *P*‐value < .05 (by BH method) was considered as the threshold.

#### 2.1.2 Analysis of the expression of FcγRs in different immune statuses of HBV‐infected patients

##### Patients

A total of 64 HBV patients (including 12 HBV infection successfully cleared (HBISC) patients, 32 CH patients and 20 CA patients) recruited from the First Bethune Hospital of Jilin University and 14 healthy individuals who attended physical examination were enrolled in this study. According to the 2009 update of AASLD^20^ and EASL guidelines,^21^ the diagnostic criteria for HBISC, CA, CH and normal groups were listed in Table 1b in details. The characteristics of patients and healthy controls were represented in Table S2. All individuals provided informed consent for using their samples for research.

##### Isolation of PBMCs and qRT‐PCR

Whole blood collection, PBMC isolation and total RNA extraction were performed as described in Section (a). Reverse transcription into cDNA was performed with PrimeScript RT Enzyme Mix (Takara). The gene expression levels were determined with SYBR PrimeScript RT‐PCR kit (Takara) by means of an Applied Biosystems 7500 fluorescent quantitative PCR system (Applied Biosystems, Foster City, CA, USA). The primer sequences (5´ to 3´) used for gene amplification were as follows: FcγR Ia forward TCACGGAAGGAGAACCTCTG and reverse CTGCTGATGTGTAGCGATGC; FcγR Ib forward GCATCGCTACACATCAGCAG and reverse CGTATTGTCACCCAGAGAACA; FcγR IIa forward ATCCCACAAGCAAACCACAG and reverse AATGACCACAGCCACAATGA; FcγR IIb forward ATCCCACAAGCAAACCACAG and reverse TACAGCAATCCCAGTGACCA; FcγR IIIIa forward TTCCCTCACAGCAAAGCAAC and reverse TGTATGGTCCTGCCTCAACC; FcγR IIIIb forward TCCTCCCAACTGCTCTGCTA and reverse AGCACGCTGTACCATTGAGG; β‐actin forward CCTAGAAGCATTTGCGGTGG and reverse GAGCTACGAGCTGCCTGACG. The relative gene expression was normalized to β‐actin and then calculated with 2^−ΔΔCt^ method. The obtained data were shown as mean ± standard deviation (SD). Significant differences between two groups were obtained when *P* < .05 which were analysed by Mann‐Whitney test by means of GraphPad Prism 5.0 software (GraphPad Prism Software, Inc., La Jolla, CA).

##### Analysis of the correlation between FcγRs expression and clinical indicators

To further investigate whether FcγRs were key regulators in different statuses of HBV infection, we analysed the correlation between the expression levels of FcγRs and clinical indicators (ALT and AST) by Spearman’s test with the same cut‐off value as described in Section (a).

#### 2.1.3 Differential expression of FcγRs on immune cells in different statuses of HBV infection

##### Subjects

Fifty‐four HBV patients (including 7 HBISC patients, 31 CH patients and 16 CA patients) and 20 healthy individuals that attended physical examination were enrolled from the First Bethune Hospital of Jilin University. As shown in Table 1c, the diagnostic and inclusion criteria for enrolling HBV‐infected patients and healthy individuals were in accordance with the description in Section (b). The clinical characteristics of patients and healthy controls are shown in Table S3. All individuals provided informed consent for using their samples for research.

##### Flow cytometry staining

Multicolour flow cytometry was used to detect FcγRs on NK cells, B cells and monocytes in different phases of HVB infection. Briefly, six freshly obtained whole blood samples were added with six groups of fluorescent marker antibodies, including CD3‐PE‐Cy7, CD56‐APC, and CD16‐FITC; CD3‐PE‐Cy7, CD19‐APC, CD5‐PE and CD32‐FITC; CD3‐APC, CD4‐PE‐Cy7 and CD8‐PE; CD14‐PE and CD16‐FITC; CD14‐PE and CD32‐FITC; and CD14‐PE and CD64‐FITC. All antibodies were purchased from BD Biosciences (San Diego, CA, USA). After incubation in the dark for 30 minutes at room temperature, the freshly obtained whole blood was treated with RBC lysis buffer (BD Biosciences) for 3 minutes in the dark. Dead cells were excluded through staining with Aqua Viability Dye (Invitrogen). The samples were detected by FACS LSR II Fortessa (BD Biosciences) and analysed with FlowJo (Tree Star, Ashland, OR, USA).

##### Analysis of the correlation between clinical indicators and FcγRs levels

The correlation between FcγRs expression on the subsets of immune cells, including CD3^−^CD56^+^CD16^+^, CD14^high^CD16^+^, CD3^−^CD19^+^CD32^+^, CD3^−^CD5^+^CD32^+^, CD3CD19^+^CD5^−^CD32^+^ and CD3^−^CD19^+^CD5^+^CD32^+^, and different clinical indicators, including ALT, AST, serum HBsAg and serum HBV DNA, was analysed by Spearman’s test. Significant *P*‐value was adjusted by BH test, and the adjusted *P*‐value < .002 indicated statistical significance.

##### Cell cytokine detection

Cytometric bead array (CBA) was used to detect cytokine levels in the plasma of subjects. Briefly, the levels of interleukin (IL)‐1β, IL‐6, IL‐10, IL‐12p70, macrophage inflammatory protein‐1 beta (MIP‐1β) and tumour necrosis factor (TNF), in plasma of patients, were measured using CBA kit (BD Biosciences) as previously described.^29^ During flow cytometry, data were acquired and analysed using Becton Dickinson CBA software.

##### Statistical analysis

The obtained data were expressed as mean ± SD, and the difference in FcγRs expression between any two groups was estimated by Mann‐Whiney *U* test within SPSS 18.0 software (SPSS, USA). Statistically significant results were obtained when *P* < .0083.

## 3 RESULTS

### 3.1 Integrative analysis of gene expression and DNA methylation profiles of HBV infection

#### 3.1.1 Identification and functional enrichment analysis of DEGs

As shown in Figure 1B. 2239, 1638, 1888, 1582 and 316 DEGs were identified in AH, CA1, CA2, CH1 and CH2 patients, respectively, as compared with healthy controls (N). Moreover, 1540, 317, 584 and 309 DEGs were identified in all C vs N, AH vs all C, AH vs CA1 and CA1 vs CH1 group, respectively. Functional enrichment analyses for DEGs between groups were performed. The results showed that DEGs significantly enriched in AH vs N group were from 18 pathways, including T cell receptor signalling, transforming growth factor‐beta (TGF‐β) signalling, leucocyte transendothelial migration, gonadotropin‐releasing hormone signalling and TLR signalling pathway. DEGs significantly enriched in all C vs N group belong to eight pathways such as T cell receptor signalling, folate biosynthesis, adherens junction, mitogen‐activated protein kinase (MAPK) signalling and TGF‐β signalling pathway.

The functional enrichment analysis was performed for DEGs between acute and chronic infection. The results showed that DEGs in AH vs all C groups were associated with various immune responses; therefore, the immune system had the most enriched genes. In addition, DEGs significantly enriched between acute and chronic infection (AH vs all C) were associated with T cell receptor signalling and leucocyte transendothelial migration pathway. In the acute phase, the T cell receptor pathway is enriched for CD3E, LCK, NFKB and so on (Figure S1). The expressions of TLR6, MYD88, TRAF6, IRF7, NFKB, MAPKs and STAT1 enriched in the Toll‐like receptor pathway were significantly higher than those in healthy controls (data not shown).

**Figure 2:**
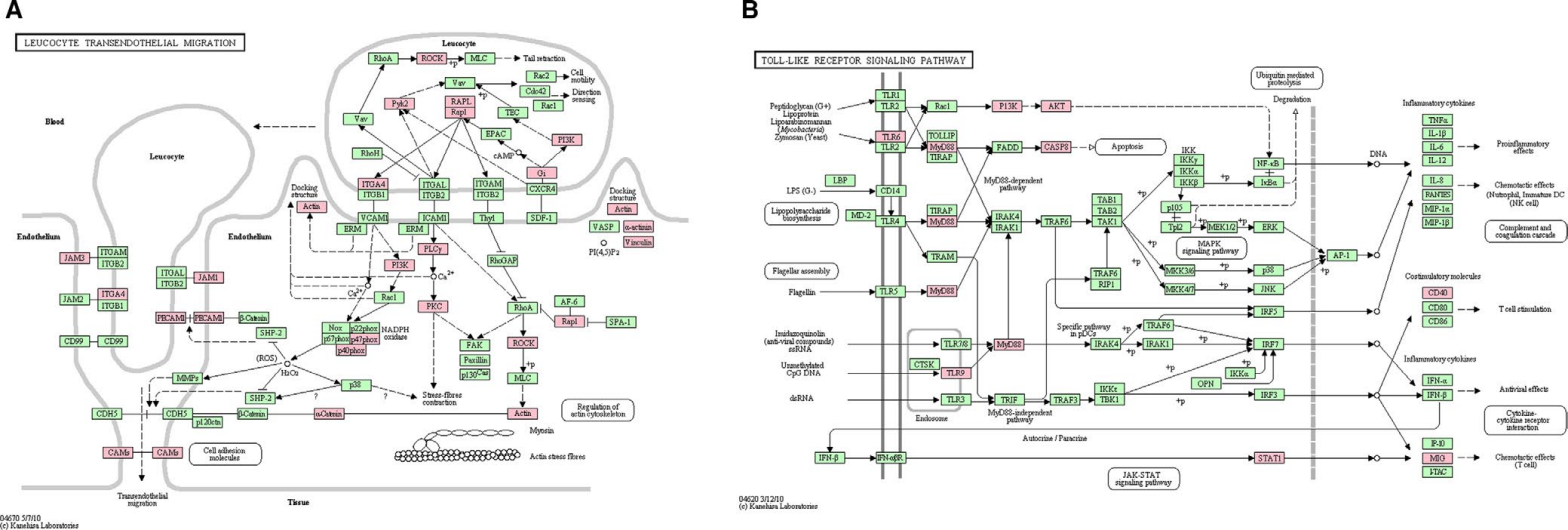
Leucocyte transendothelial migration pathway (A) and Toll‐like receptor signalling pathway (B) enriched by differentially expressed genes between acute and chronic hepatitis B virus (HBV) infection. Pink nodes indicate differentially expressed genes

As shown in Figure 2A, nine DEGs up‐regulated in AH vs all C were enriched in leucocyte transendothelial migration pathway, including integrin subunit beta 1 (ITGB1), integrin subunit alpha 4 (ITGA4), actin beta (ACTB), vinculin (VCL), myosin light chain 9 (MYL9), neutrophil cytosolic factor 1 (NCF1), NCF4, phosphoinositide‐3‐kinase regulatory subunit 5 (PIK3R5) and cell division cycle 42 (CDC42).

We performed functional enrichment analysis for DEGs in AH vs CA1 group. Among these DEGs, interferon‐stimulated genes (ISGs), including interferon‐induced protein with tetratricopeptide repeats 3 (IFIT3), interferon‐alpha inducible protein 27 (IFI27), IFI30 and IFI35, interferon‐induced transmembrane protein 3 (IFITM3) and FcγRs, such as FcγR Ib, FcγR Ia and FcγR IIa, showed significant differential expression. These DEGs were significantly enriched in functions associated with immune response and in key pathways such as TLR signalling pathway. As shown in Figure 2B, DEGs such as TLR6, TLR9, signal transducer and activator of transcription 1 (STAT1) and myeloid differentiation primary response 88 (MYD88) were enriched in TLR signalling pathway.

The functional enrichment analysis was also performed for DEGs from CA1 and CH1 phases of chronic hepatitis B. We found that 309 DEGs significantly enriched between CA1 and CH1 exhibited important functions related to inflammatory process. Of these, expression levels of IFI27 and FcγRs (including FcγR Ib, FcγR Ia and FcγR IIIa) were markedly higher in CH1 patients as compared with CA1 patients.

#### 3.1.2 Analysis of the correlation between clinical indicators and gene expression levels

**Figure 3:**
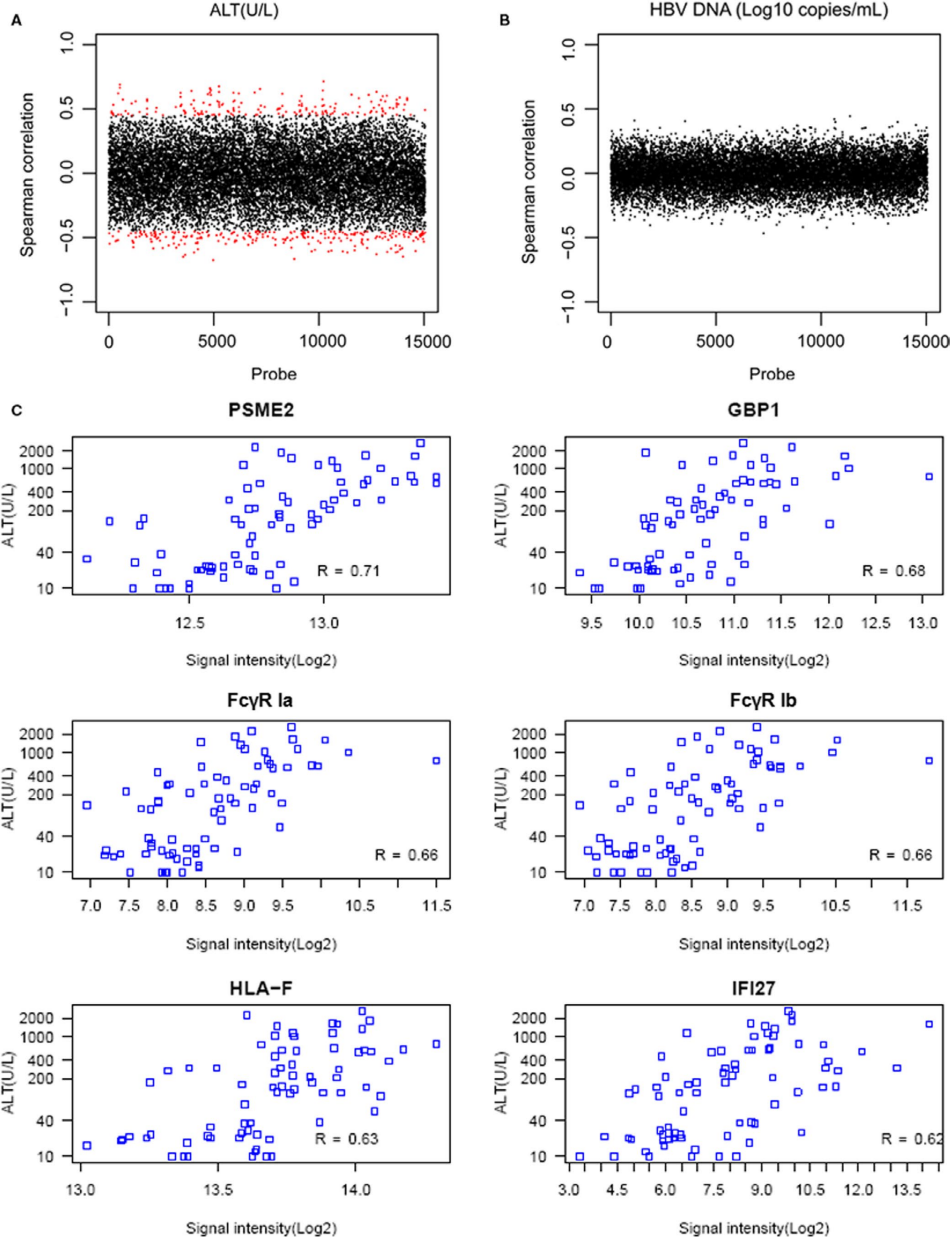
Analysis of the correlation between clinical indicators and gene expression levels. A, The correlation between alanine aminotransferase (ALT) levels and gene expression levels. B, The correlation between HBV DNA and gene expression levels. C, Key differentially expressed genes associated with ALT levels

With the cut‐off value of adjusted *P*‐value < .05 and |spearman coefficient| > 0.45, a significant correlation existed between ALT and gene expression levels (Figure 3A), but no genes were markedly correlated with HBV DNA (Figure 3B). A total of 397 genes showed closed correlation with ALT level, including 161 positively correlated genes and 236 negatively correlated genes. Functional enrichment analyses showed that these genes were significantly enriched in immune response, antigen processing and presentation, major histocompatibility complex (MHC) class I receptor activity and proteasome activity. According to Spearman’s coefficient, several key DEGs, including proteasome activator subunit 2 (PSME2), guanylate‐binding protein 1 (GBP1), FcγR Ia, FcγR Ib, HLA‐F and IFI27, correlated with ALT level (Figure 3C).

#### 3.1.3 Identification of DMGs and functional enrichment analysis

The main purpose of methylation profiling studies is to observe the DNA methylation status of peripheral blood in HBV infection patients and to identify DMG related DEGs. Based on the platform of Roche NimbleGen Human DNA Methylation 3x720K CpG Island Plus RefSeq Promoter Array, 711 794 probes corresponding to 43 062 transcripts and 24 928 known genes were obtained. After probe filtration and quality assessment, 62 samples (7 AH, 15 CA1, 12 CA2, 12 CH1, 9 CH2 and 7 N) were selected for the development of DNA methylation profiles, including 564 464 probes corresponding to 17 731 genes. Using *t* test, 4867 (819 up‐regulated and 4048 down‐regulated), 4248 (591 up‐regulated and 3657 down‐regulated) and 1703 (601 up‐regulated and 1102 down‐regulated) DMGs were identified in AH vs N, AH vs all C and all C vs N groups, respectively.

The functional enrichment analysis for DMGs in AH vs N group showed that these significantly enriched DMGs were associated with signal transduction, olfactory transduction, cyanoamino acid metabolism and intercellular communication. DMGs significantly enriched in all C vs N group were related to focal adhesion, tight junction, Janus kinase‐STAT signalling, calcium signalling and Wnt signalling pathway. DMGs significantly enriched in all C vs N group belong to perception of chemical stimuli, perception of odour, response to stimuli, differentiation of keratinocytes, G protein‐coupled receptor signalling pathway and inflammatory response. The results suggested that extensive methylation occurs during both acute and chronic HBV infection. In the acute HBV infection, a large number of genes involved in the immune response and T cell receptor complex formation are abnormally methylated. While, in the chronic HBV infection, cancer‐related genes, especially MSCTP1, RASSF1A and CDKN2A, which are reported in liver cancer, are abnormally methylated, suggesting that accumulation of abnormal methylation events has begun in the stage of chronic HBV infection.

#### 3.1.4 Integrative analysis of gene expression and DNA methylation profiles

In combination with gene expression profile data, 478 and 2097 genes were identified from 819 up‐regulated and 4048 down‐regulated DMGs in AH vs N group, respectively. Among 478 up‐regulated DMGs, 59 genes showed down‐regulated expression and were significantly enriched in important functions associated with RNA metabolism and cell movement. Among 2097 down‐regulated DMGs, 207 genes showed up‐regulated expression and were markedly enriched in key functions related to immune response, regulation of immune response and glycerol lipid metabolism. Five lowly methylated and highly expressed genes were found to be enriched in T cell receptor signalling pathway, including nuclear factor kappa B subunit 2 (NFκB2), cytotoxic T lymphocyte‐associated protein 4 (CTLA4), CD3e molecule (CD3E), mitogen‐activated protein kinase 3 (MAPK3) and LCK proto‐oncogene, Src family tyrosine kinase (LCK) (Figure S1).

In addition, 38 lowly methylated and highly expressed genes and 12 highly methylated and lowly expressed genes were identified in all C vs N group in combination with gene expression profile data. These highly methylated and lowly expressed genes, such as Ras association domain family member 1 (*RASSF1A*) and cyclin‐dependent kinase inhibitor 2A (*CDKN2A*), were predominantly enriched in multiple cancer‐associated signalling pathways.

In addition, 40 lowly methylated and highly expressed genes and five highly methylated and lowly expressed genes were identified in AH vs all C groups by integrative analysis of gene expression and DNA methylation profiles. Of these, 40 lowly methylated and highly expressed genes, seven were associated with immune response, including complement C1q B chain (C1QB), CTLA4, leucocyte immunoglobulin‐like receptor A1 (LILRA1), leucocyte immunoglobulin‐like receptor B1 (LILRB1), LIM domain only 2 (LMO2), NCF1 and NCF4.

### 3.2 Analysis of FcγRs expression in different immune statuses of chronic HBV‐infected patients

#### 3.2.1 The expression of FcγRs in different statuses of chronic HBV‐infected patients

**Figure 4:**
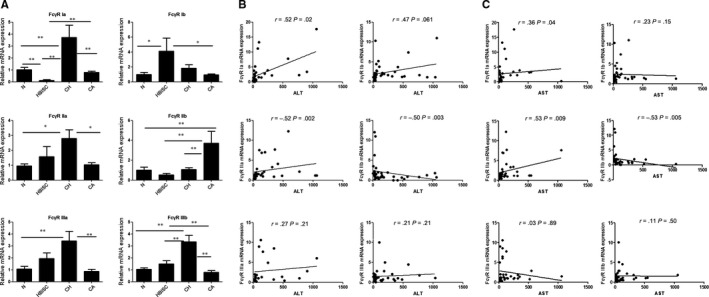
The expression of FcγRs in different immune state of chronic HBV infection and analysis of the correlation between clinical indicators and FcγR expression levels. A, The expression levels of FcγRs. B, The correlation between ALT levels and FcγR expression levels. C, The correlation between aspartate transaminase (AST) and FcγR expression levels. Data were expressed as mean ± standard deviation. *P < .05 and **P < .01

We performed qRT‐PCR analysis to detect FcγRs expression in chronic HBV infection. As shown in Figure 4A, the expression levels of FcγR Ia, FcγR IIa, FcγR IIIa and FcγR IIIb in CH patients were increased as compared to CA patients and healthy control (all *P* < .05). Moreover, FcγR Ia and FcγR IIb expressions in CA group were markedly increased as compared with that of HBISC group, while FcγR Ib and FcγR IIIb expression was decreased in CA group (all *P* < .05). FcγR IIb expression was higher in CA group relative to that of CH group (*P* < .01).

#### 3.2.2 Analysis of the correlation between clinical indicators and FcγR expression levels

The correlation between FcγR expression and clinical indicators, including ALT and AST, was analysed. As shown in Figure 4B,C, ALT and AST showed a positive correlation with the expression of activated receptors such as FcγR Ia and FcγR IIa and negative correlation with the expression of inhibitory FcγR IIb, indicating that the differential expression of FcγR Ia, FcγR IIa and FcγR IIb may be used to distinguish between different immune statuses of HBV infection.

### 3.3 Differential expression of FcγRs on immune cells in different statuses of HBV infection

#### 3.3.1 Differential expression of FcγRs on immune cells in HBV‐infected patients

**Figure 5:**
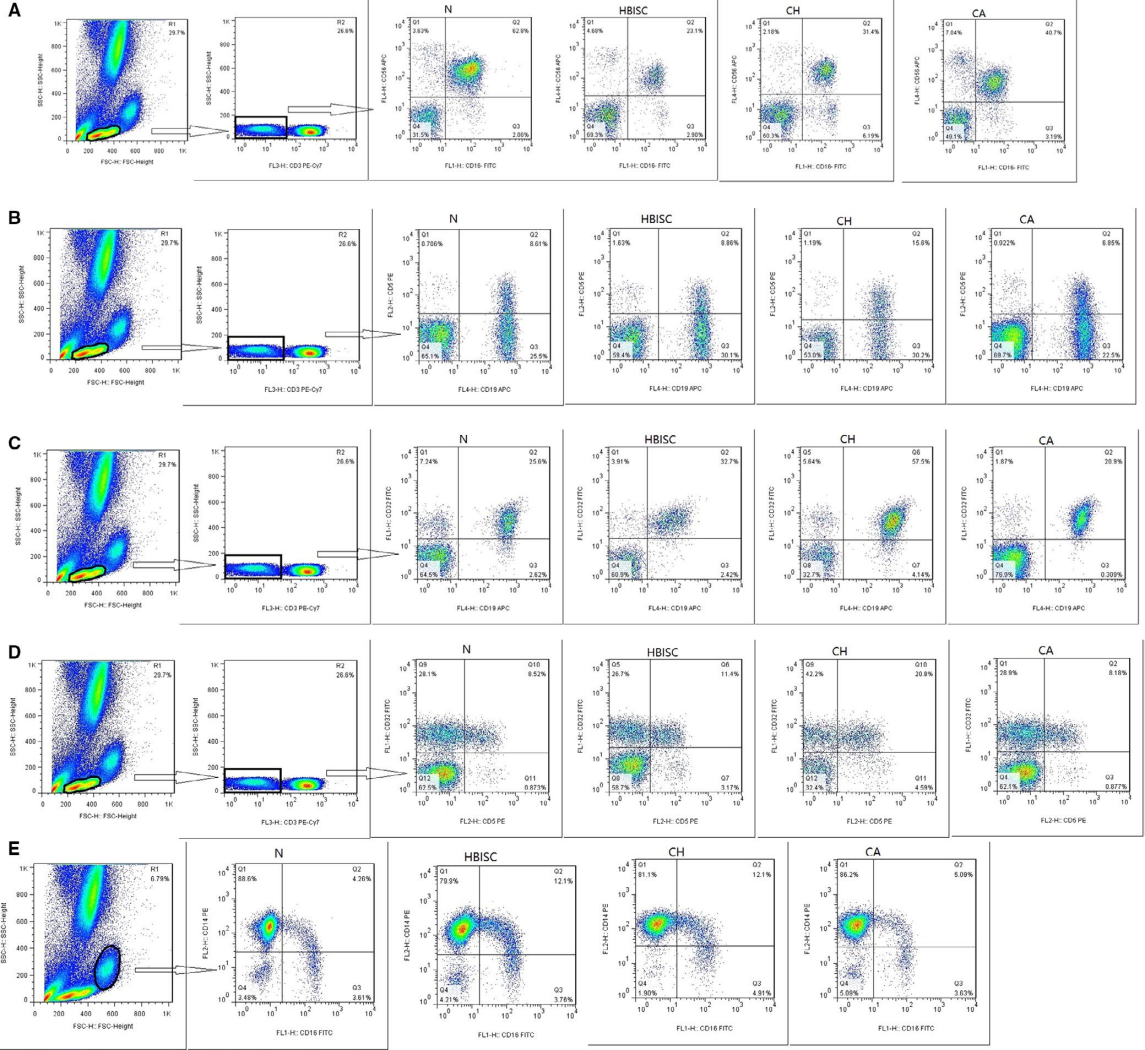
The expressions of FcγRs on immune cells in different state of HBV infection. A, Different expression of FcγRs on the subsets of CD3‐CD56+CD16+NK cells between four groups. B, Different expression of FcγRs on the subsets of CD3‐CD19+CD5‐ and CD3‐CD19+CD5+B cells between four groups. C, Different expression of FcγRs on the subsets of CD3‐CD19+CD32+B cells between four groups. D, Different expression of FcγRs on the subsets of CD3‐CD5+CD32+B cells between four groups. E, Different expression of FcγRs on the subsets of CD14highCD16+ monocytes between four groups. One or two columns on the left show a schematic diagram of cell subsets in a flow cytometry experiment

The expression of FcγR III (CD16) on NK cells and their subsets in patients with different HBV infection was detected. As shown in Figure 5A and Table S4A, total NK and CD3^−^CD56^+^CD16^+^ NK cells in CH patients significantly decreased as compared with those in CA patients and healthy controls (all *P* ≤ .001). In addition, total NK and CD3^−^CD56^+^CD16^+^ NK cells in HBISC patients decreased in HBISC patients as compared with those in healthy controls (*P* = .002). There were no significant differences in CD3^−^CD56^−^CD16^+^ and CD3^−^CD56^+^CD16^−^ NK cells between any two groups.

The FcγR II (CD32) expression on B cells and their subsets in different HBV‐infected patients was detected. As shown in Figure 5B,5 and 5 and Table S4B, CD3^−^CD5^+^, CD3^−^CD19^+^CD5^+^, CD3^−^CD19^+^CD32^+^, CD3^−^CD5^+^CD32^+^ and CD3^−^CD19^+^CD5^+^CD32^+^B cells in CH patients were higher than those in healthy controls (*P* = .005, .006, .008, .002 and .006, respectively). CD3^−^CD19^+^CD5^−^, CD3^−^D19^+^CD32^+^ and CD3^−^CD19^+^CD5^−^CD32^+^B cells in CH patients were markedly increased as compared with those in CA patients (*P* = .006, .008 and .004, respectively). No significant differences were observed in CD3^−^CD19^+^, CD3^−^CD5^+^, CD3^−^CD19^+^CD5^−^ and CD3^−^CD19^+^CD5^+^ B cells between any two groups.

The expression of FcγRs on monocyte cells was also detected. As indicated in Figure 1E and Table S4C, CD14^high^CD16^+^ cells in CH patients were increased in comparison with those in CA patients and healthy controls (*P* < .001). Moreover, CD14^high^CD16^+^ cells were markedly increased in HBISC patients as compared with those in healthy controls (*P* = .002). No significant differences were observed in CD14^+^CD32^+^ and CD14^+^CD64^+^ cells between any two groups.

#### 3.3.2 Analysis of the correlation between clinical indicators and FcγR expression levels

**Figure 6:**
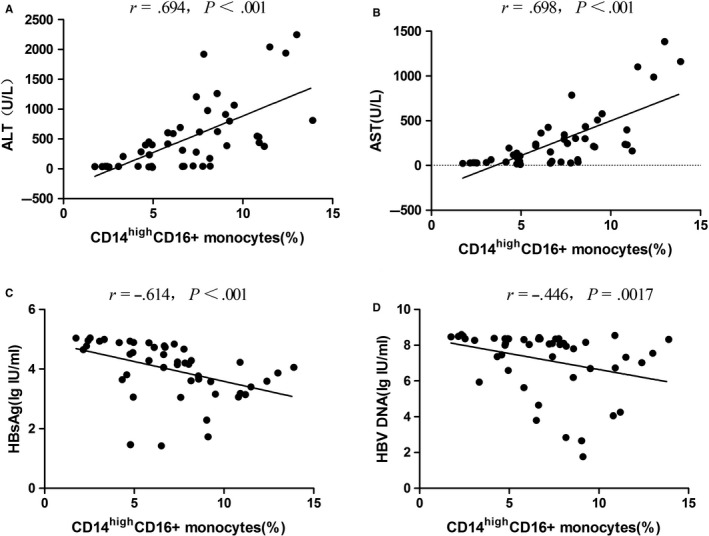
The correlation between FcγR expression on CD14^high^CD16^+^ monocytes and different clinical indicators, including ALT, AST, serum HBsAg and serum HBV DNA

The correlation between FcγR expression on the subsets of immune cells, including CD3^−^CD56^+^CD16^+^, CD14^high^CD16^+^, CD3^−^CD19^+^CD32^+^, CD3^−^CD5^+^CD32^+^, CD3^−^CD19^+^CD5^−^CD32^+^ and CD3^−^CD19^+^CD5^+^CD32^+^, and different clinical indicators, including ALT, AST, serum HBsAg and serum HBV DNA, was analysed by Spearman’s test. As shown in Figure 6, only CD14^high^CD16^+^ monocyte cells showed a positive correlation with ALT (r = 0.694, *P* < .001) as well as AST (*r* = .698, *P* < .001) and negative correlation with serum HBsAg (*r* = −.614, *P* = .0017) and serum HBV DNA (*r* = −.446, *P* = .0017). There was no significant correlation between CD3^−^CD56^+^CD16^+^, CD3^−^CD19^+^CD32^+^, CD3^−^CD5^+^CD32^+^, CD3^−^CD19^+^CD5^−^CD32^+^ and CD3^−^CD19^+^CD5^+^CD32^+^ and different clinical indicators (*P* > .002).

#### 3.3.3 Analysis of cytokine levels in plasma of HBV‐infected patients

**Figure 7:**
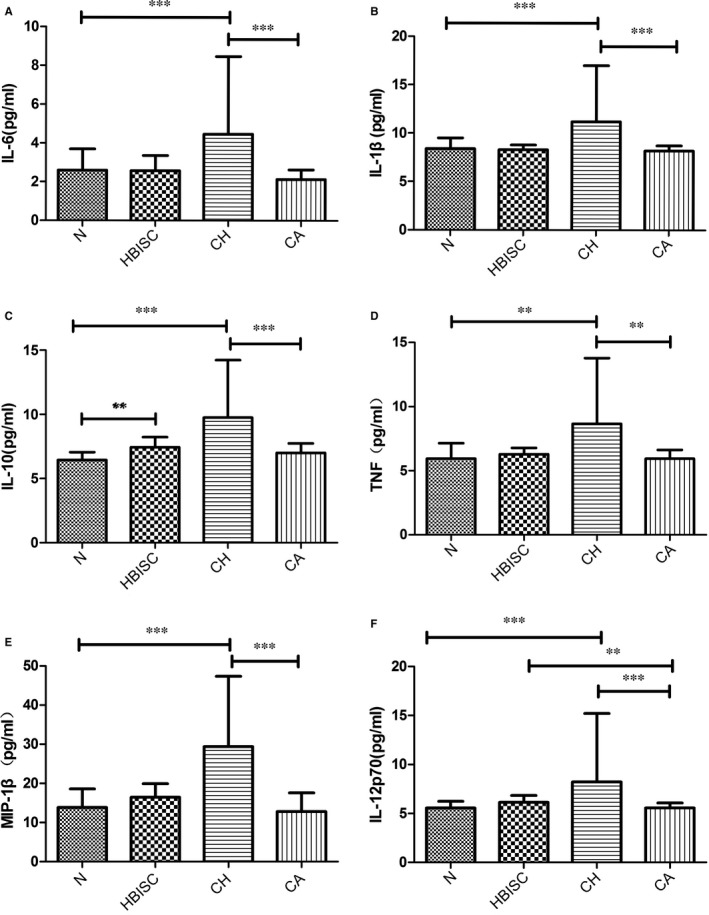
The levels of key cytokines (IL‐6, IL‐1β, IL‐10, TNF, MIP‐1β and IL‐12p70) in plasma of patients with different immune statuses of HBV infection. Data were expressed as mean ± standard deviation. *P < .05, **P < .01 and ***P < .001

Cytokine levels in plasma of HBV‐infected patients were analysed. We found that levels of IL‐6, IL‐1β, IL‐10, TNF, MIP‐1β and IL‐12p70 were markedly increased in CH patients relative to those in CA patients and healthy controls (all *P* ≤ .001, Figure 7). Moreover, IL‐10 and IL‐12p70 levels in HBISC patients were obviously higher than those in healthy controls (*P* = .002 and .007, respectively) (Figure 7).

## 4 DISCUSSION

The present study investigated key genes and pathways associated with different immune statuses of HBV infection. The results showed that TLR signalling pathway and leucocyte transendothelial migration pathway (including ITGB1) were enriched during acute HBV infection. Key DEGs such as FcγR Ia, FcγR Ib and IFI27 showed differential expression between AH and CA1 phases and between CA1 and CH1 phases. Furthermore, the differential expression of FcγR Ia, FcγR IIa and FcγR IIb was identified between CA and CH phases by experimental validation. Integrative analysis of gene expression and DNA methylation profile showed that lowly methylated and highly expressed genes (such as CTLA4 and MAPK3) were enriched in T cell receptor signalling pathway during acute HBV infection. Highly methylated and lowly expressed genes (including RASSF1A and CDKN2A) were identified in chronic HBV infection.

During the HBV infection, the innate immune response inhibits the replication and the spread of HBV in the early stage, while the adaptive HBV‐specific immune response primarily plays a role in viral clearance in the late stage.^14^ In acute HBV infection, the immune response to clear the virus precedes the clinical features such as jaundice and elevated transaminases. Actually, it is difficult to collect samples of acute HBV infection at the window period. Stefan et al reported that they did not detect natural immune response in the early stage in the acute infection of the apes, but they emphasized that the adaptive immune response played a role in clearing the virus after a few weeks.^30^ Besides, there were increasing evidence showed that HBV inhibits natural immune response by regulating Toll‐like receptors on the surface of immune cells.^31^, ^32^, ^33^ In our study, the key different pathway between AH and all C groups was Toll‐like receptor pathway also known as TLR signalling pathway. The innate immune response mediated by TLR signalling pathway is found to play a role in the control of HBV infection.^17^ HBeAg induces the pathogenesis of HBV infection through targeting TLR‐mediated signalling pathways to evade innate immune responses.^34^ Moreover, higher level of HBsAg may attenuate TLR‐mediated immune responses to evade innate and adaptive immune responses and maintain persistent HBV infection.^35^ In our study, TLR signalling pathway was significantly enriched during acute infection of HBV. Thus, innate immune responses mediated by TLR signalling pathway may regulate the acute phase of HBV infection. In addition, leucocyte transendothelial migration is a key process to evoke the innate or adaptive immune response.^36^ ITGB1 haplotype is shown to be correlated with the clearance of HBV infection.^37^ Our results showed that leucocyte transendothelial migration pathway was enriched during AH phase of HBV infection, in which ITGB1 was included. Therefore, we speculate that leucocyte transendothelial migration pathway may be involved in HBV clearance during acute infection via ITGB1 regulation.

HBV‐specific T cell dysfunction and decreased T cell numbers contribute to the chronic of HBV infection.^10^ In our study, DEGs between AH and N, AH and chronic of HBV infection were also enriched in the T cell receptor signalling. Almost no type I IFN response was detected during the entire acute infection process, and NK cell activation and function were inhibited at the peak of viraemia.^38^ Combined with our findings, T cell‐mediated adaptive immune responses are predominantly dominant during acute HBV infection. The maturation and activation of T cells are mediated by immune synapses formed by the binding of T cell receptors, MHC and other T cell receptor accessory molecules.^39^ Among them, CD3E, CD4 and LCK are auxiliary molecules of important T cell receptors that form this immune synapse. We observed that these genes are highly expressed in the acute phase and low in the chronic phase and healthy controls, suggesting that the activation status of these genes is related to the natural course of disease after HBV infection. Furthermore, lowly methylated and highly expressed genes (including *CTLA4* and *MAPK3*) in AH phase were enriched in T cell receptor signalling pathway by integrative analysis of DNA methylation profile. It is reported that defective antigen presentation may induce T cell tolerance by combining CTLA4 and PD 1.^40^ DNA immunization with the fusion of CTLA‐4 to HBV core protein has the ability to promote Th2‐type responses for HBV elimination.^41^ In addition, MAPK3 is important for the induction of T cell energy and has the capacity to activate T cell responses via dendritic cells in autoimmunity.^42^ Based on our results, we suggest that the epigenetic regulation of *CTLA4* and *MAPK3* by DNA methylation may be involved in the acute phase of HBV infection via regulation of T cell receptor signalling pathway. In addition, highly methylated and lowly expressed genes (including *RASSF1A* and *CDKN2A*) were identified in chronic HBV infection. It has been confirmed that the reduced expression and hypermethylation of *RASSF1A* were frequently observed in HCC and played crucial roles in HCC tumorigenesis and metastases.^43^ The combination of serum RASSF1A methylation and α‐fetoprotein level is suggested as a promising biomarker to discriminate HCC patients with chronic HBV infection.^44^ Moreover, *CDKN2A* promoter methylation and lack of p16 expression are main hallmarks for the differentiation of HCC from other liver tumours.^45^ Taken together, we speculate that the methylation of *RASSF1A* and *CDKN2A* may exhibit an important role in chronic HBV infection and HBV‐related liver diseases.

FcγRs is a receptor for the Fc segment of immunoglobulin IgG. It is known that FcγRs mediate multiple immunological functions, which influence both innate and adaptive responses.^46^ Besides, FcγRs‐mediated antibody effector functions are compromised or suppressed during persistent viral infection.^47^, ^48^ FcγRs are classified into CD64 (FcγR I), CD32 (FcγR II) and CD16 (FcyR III) depending on their affinity and structure. Among them, FcγR I show the stronger affinity to IgG Fc than that of FcγR II and FcγR III. NK cells recruit immune cells to the site of infection by killing immature dendritic cells and secreting inflammatory cytokines and chemokines.^49^ CD3‐CD56+CD16+ NK cells can activate antiviral‐infected cells. When the Fc segment of an antibody is conjugated to FcγR III, it is linked to the ζ dimer contained in NK cells and then transmits an activation signal to the cells. Subsequently, antibody‐dependent cytotoxicity is performed to destroy target cells. FcγR IIb, which is the only Fcγ family receptor on the surface of B cell, is a negative regulator of innate immunity and acquired immunity.^50^, ^51^ The CD3‐CD19+CD32+ B cells in the CH group were significantly higher than those in the CA and N groups, suggesting that the immune system controlled the excessive activation and proliferation of B cells by the increased CD32+ B cells in the immune‐clearance period of chronic hepatitis B. In our study, FcγR Ia and FcγR Ib were differentially expressed between AH and CA1 phases as well as CA1 and CH1 phases. Moreover, experimental validation confirmed the differential expression of FcγR Ia, FcγR IIa and FcγR IIb between CA and CH phases. ALT and AST showed a positive correlation with the expression of activated receptors such as FcγR Ia and FcγR IIa and negative correlation with the expression of inhibitory FcγR IIb. These findings suggest the use of FcγR Ia, FcγR IIa and FcγR IIb to distinguish the different immune statuses of HBV infection and as potential targets for immunotherapy of HBV‐related liver diseases.

There was significantly difference of DMGs between acute and chronic HBV infection and healthy controls, indicating that the HBV infection indeed leads to epigenetic recombination of host genes. Based on the comprehensive analysis of DEGs and DMGs, hypermethylated genes (low expressed) or demethylated genes (high expressed) were mainly enriched in the immune response and T cell receptor pathways. The T cell receptor (TCR) ‐CD3 complex formed by CD3E and TCR plays a key role in antigen recognition, linking downstream signalling pathways and T cell maturation.^52^ Besides, the mutation or non‐expression of CD3E is closely related to immunodeficiency.^53^ LCK belongs to the Src family of tyrosine protein kinases, which are involved in T cell maturation and proliferation, and mediate the key downstream molecules of MAPK and NFKB through TCR‐CD3 complex.^54^, ^55^ CD3E and LCK are upstream molecules of the T cell receptor pathway. In the acute HBV infection, hypomethylated and high expressed CD3E and LCK activates the T cell response process. Our results suggested that the T cell receptor pathway is regulated by epigenetics in the acute infection, especially in the process of T cell receptor complex formation. The methylation status of the enriched gene in T cell receptor pathway is closely related to their expression levels. In addition, other DMGs such as MAPK3 and NFKB2, which are downstream of the T cell receptor pathway, are also fast transcription factors that mediate inflammation and immune response processes.

Clinically, elevated ALT is used as a marker of immune activation and as a basis for immune staging and treatment.^56^, ^57^ Our results suggested that genes associated with ALT levels were mainly enriched in immune responses, antigen processing and presentation, MHC class I receptor activity a and proteasome activities. Functional enrichment and Spearman’s correlation analysis showed that the expressions of HLA‐F, IFI27 and PSME2 were associated with ALT. Among them, HLA‐F acts as a surface marker for activated lymphocytes, whereas PSME2 (also known as PA28β) is associated with maturation of dendritic cells and MHC class I antigen presentation.^58^, ^59^ Immune cells recognize viruses and secrete IFN through pattern recognition receptors (PRRs), which induce the production of a series of Interferon‐stimulated genes (ISGs).^60^ The primary role of ISGs is to amplify the IFN signalling pathway, induce the production of cytokines capable of activating adaptive immune responses and directly inhibit the virus.^61^ Our results showed that there were differential expressions of ISGs between AH and all C groups, as well as between different stages of chronic infection, such as IFI27, IFI30, IFI35, IFIT3 and IFITM3. Among of them, the FC of IFI27 was the highest with FC=10.44 and *P* = .0014225. In the early stages of viral infection, IFITM3 inhibits virus entry into cells, whereas IFI27 is produced in the late stages of viral infection, mediating apoptosis by disrupting mitochondrial membrane stability.^62^, ^63^ IFI30 is induced by type II IFN and mainly assists in the antigen presentation process restricted by MHC class II antigens.^64^

We identified the DEGs and DGMs in HBV infection and predicted the possible roles by enrichment analysis. One limitation in this study was that we did not validate the roles of DEGs and DMGs on the HBV infection. However, in the next study, we will collect peripheral blood PBMC from patients with different HBV infection status, and the expression levels of main DEGs will be detected by qRT‐PCR and Western blot. In addition, the mechanisms related to DEGs and DGMS in different HBV infection states will be further studied.

In conclusion, our study reveals key genes and pathways that may be used to distinguish between different immune statuses of HBV infection. The innate immune response mediated via TLR signalling pathway may regulate the acute phase of HBV infection. Leucocyte transendothelial migration pathway may be involved in HBV clearance during acute infection via ITGB1 regulation. In addition, FcγR Ia, FcγR IIa and FcγR IIb may be used to distinguish different immune statuses of HBV infection and serve as potential targets in immunotherapy of HBV‐related liver diseases. The epigenetic regulation of *CTLA4* and *MAPK3* by DNA methylation may be involved in the acute phase of HBV infection through regulating T cell receptor signalling pathway, while the methylation of *RASSF1A* and *CDKN2A* may exhibit an important role in chronic HBV infection.

## CONFLICT OF INTEREST

The authors declare that they have no competing interests.

## AUTHORS’ CONTRIBUTIONS

Jinglan Jin and Hongqin Xu involved in conception and design of the research; Ruihong Wu and Na Gao involved in the acquisition of data; Na Wu and Shibo Li performed this study; Jinglan Jin and Junqi Niu involved in analysis and interpretation of data; Jinglan Jin and Junqi Niu drafted the manuscript. All authors read and approve final manuscript.

## Data Availability

The datasets used and/or analysed during the current study are available from the corresponding author on reasonable request.

## References

[1] Ott JJ , Stevens GA , Groeger J , Wiersma ST . Global epidemiology of hepatitis B virus infection: new estimates of age‐specific HBsAg seroprevalence and endemicity. Vaccine. 2012;30:2212–2219. 22273662 10.1016/j.vaccine.2011.12.116

[2] Kao J‐H , Chen D‐S . Global control of hepatitis B virus infection. Lancet Infect Dis. 2002;2:395–403. 12127351 10.1016/s1473-3099(02)00315-8

[3] Lavanchy D . Worldwide epidemiology of HBV infection, disease burden, and vaccine prevention. J Clin Virol. 2005;34(Suppl 1):S1–S3. 10.1016/s1386-6532(05)00384-7 16461208

[4] Seeger C , Mason WS . Hepatitis B virus biology. Microbiol Mol Biol Rev. 2000;64:51–68. 10704474 10.1128/mmbr.64.1.51-68.2000 PMC98986

[5] Goldstein ST , Zhou F , Hadler SC , Bell BP , Mast EE , Margolis HS . A mathematical model to estimate global hepatitis B disease burden and vaccination impact. Int J Epidemiol. 2005;34:1329–1339. 16249217 10.1093/ije/dyi206

[6] Ott JJ , Ullrich A , Mascarenhas M , Stevens GA . Global cancer incidence and mortality caused by behavior and infection. Journal of Public Health. 2010;33:223–233. 20935133 10.1093/pubmed/fdq076

[7] Bosetti C , Turati F , La Vecchia C . Hepatocellular carcinoma epidemiology. Best Pract Res Clin Gastroenterol. 2014;28:753–770. 25260306 10.1016/j.bpg.2014.08.007

[8] Greten TF , Greten TF , Korangy F . Immunotherapy of hepatocellular carcinoma. J Hepatol. 2006;45(6):868–878. 17046096 10.1016/j.jhep.2006.09.004

[9] Boeijen LL , Hoogeveen RC , Boonstra A , Lauer GM . Hepatitis B virus infection and the immune response: The big questions. Best Pract Res Clin Gastroenterol. 2017. 10.1016/j.bpg.2017.05.003 28774408

[10] Fisicaro P , Valdatta C , Boni C , et al. Early kinetics of innate and adaptive immune responses during hepatitis B virus infection. Gut. 2009;58:974–982. 19201769 10.1136/gut.2008.163600

[11] Akira S , Uematsu S , Takeuchi O . Pathogen recognition and innate immunity. Cell. 2006;124:783–801. 16497588 10.1016/j.cell.2006.02.015

[12] Maini MK , Gehring AJ . The role of innate immunity in the immunopathology and treatment of HBV infection. J Hepatol. 2016;64:S60–S70. 27084038 10.1016/j.jhep.2016.01.028

[13] Webster GJ , Reignat S , Maini MK , et al. Incubation phase of acute hepatitis B in man: dynamic of cellular immune mechanisms. Hepatology. 2000;32:1117–1124. 11050064 10.1053/jhep.2000.19324

[14] Bertoletti A , Ferrari C . Innate and adaptive immune responses in chronic hepatitis B virus infections: towards restoration of immune control of viral infection. Gut. 2011;61(12):1754–1764. 22157327 10.1136/gutjnl-2011-301073

[15] Bertoletti A , Ferrari C . Adaptive immunity in HBV infection. J Hepatol. 2016;64:S71–S83. 27084039 10.1016/j.jhep.2016.01.026

[16] Wei C , Ni C , Song T , et al. The hepatitis B virus X protein disrupts innate immunity by downregulating mitochondrial antiviral signaling protein. J Immunol. 2010;185:1158–1168. 20554965 10.4049/jimmunol.0903874

[17] Zhang E , Lu M . Toll‐like receptor (TLR)‐mediated innate immune responses in the control of hepatitis B virus (HBV) infection. Med Microbiol Immunol. 2015;204:11–20. 25550115 10.1007/s00430-014-0370-1 PMC4305100

[18] Zeybel M , Vatansever S , Hardy T , et al. DNA methylation profiling identifies novel markers of progression in hepatitis B‐related chronic liver disease. Clin Epigenet. 2016;8:48. 10.1186/s13148-016-0218-1 PMC4857425 27152124

[19] Zou Q , Wen W , Zhang X . Presepsin as a novel sepsis biomarker. World J Emerg Med. 2014;5:16–19. 25215141 10.5847/wjem.j.issn.1920-8642.2014.01.002 PMC4129857

[20] Lok AS , McMahon BJ . Chronic hepatitis B: update 2009. Hepatology. 2009;50:661–662. 19714720 10.1002/hep.23190

[21] Conjeevaram HS , Lok AS‐F . Management of chronic hepatitis B. J Hepatol. 2003;38:90–103. 10.1016/s0168-8278(02)00431-2 12591188

[22] Shaoji Y , Hong R .Lemology: People's Medical Publishing House; 2008.

[23] Smyth GK , Ritchie, M , Thorne, N , et al. Limma: Linear models for microarray data In: Gentleman R, Carey V, Huber W, Irizarry R, Dudoit S, eds. Bioinformatics and computational biology solutions using R and bioconductor. Statistics for biology and health. New York, NY: Springer; 2005:397–420.

[24] CNVassoc .Corrected p values using Benjamini & Hochberg approach.

[25] Ashburner M , Ball CA , Blake JA , et al. Gene Ontology: tool for the unification of biology. Nat Genet. 2000;25:25–29. 10802651 10.1038/75556 PMC3037419

[26] Kanehisa M , Goto S . KEGG: kyoto encyclopedia of genes and genomes. Nucleic Acids Res. 2000;28:27–30. 10592173 10.1093/nar/28.1.27 PMC102409

[27] Morris MR , Ricketts CJ , Gentle D , et al. Genome‐wide methylation analysis identifies epigenetically inactivated candidate tumour suppressor genes in renal cell carcinoma. Oncogene. 2011;30:1390. 21132003 10.1038/onc.2010.525

[28] Roche.NimbleGen . Arrays User's Guide DNA Methylation Arrays Version 7.2. 2010.

[29] Fujii N , Hiraki A , Aoe K , et al. Serum cytokine concentrations and acute graft‐versus‐host disease after allogeneic peripheral blood stem cell transplantation: concurrent measurement of ten cytokines and their respective ratios using cytometric bead array. Int J Mol Med. 2006;17:881–885. 16596275

[30] Schaefer S . Hepatitis B virus taxonomy and hepatitis B virus genotypes. World J Gastroenterol. 2007;13:14. 17206751 10.3748/wjg.v13.i1.14 PMC4065870

[31] Arpaia N , Barton GM . Toll‐like receptors: key players in antiviral immunity. Curr Opin Virol. 2011;1:447–454. 22440908 10.1016/j.coviro.2011.10.006 PMC3311989

[32] Medzhitov R . Toll‐like receptors and innate immunity. Nat Rev Immunol. 2001;1:135. 11905821 10.1038/35100529

[33] Kawai T , Akira S . The role of pattern‐recognition receptors in innate immunity: update on Toll‐like receptors. Nat Immunol. 2010;11:373. 20404851 10.1038/ni.1863

[34] Lang T , Lo C , Skinner N , Locarnini S , Visvanathan K , Mansell A . The hepatitis B e antigen (HBeAg) targets and suppresses activation of the toll‐like receptor signaling pathway. J Hepatol. 2011;55:762–769. 21334391 10.1016/j.jhep.2010.12.042

[35] Jiang M , Broering R , Trippler M , et al. Toll‐like receptor‐mediated immune responses are attenuated in the presence of high levels of hepatitis B virus surface antigen. J Viral Hepatitis. 2014;21:860–872. 10.1111/jvh.12216 24498958

[36] Muller WA . Mechanisms of leukocyte transendothelial migration. Annu Rev Pathol. 2011;6:323–344. 21073340 10.1146/annurev-pathol-011110-130224 PMC3628537

[37] Park T‐J , Chun J‐Y , Bae J‐S , et al. Putative Association of ITGB1 Haplotype with the Clearance of HBV Infection. Genomics Inform. 2010;8:9–18.

[38] Dunn C , Peppa D , Khanna P , et al. Temporal analysis of early immune responses in patients with acute hepatitis B virus infection. Gastroenterology. 2009;137:1289–1300. 19591831 10.1053/j.gastro.2009.06.054

[39] Grakoui A , Bromley SK , Sumen C , et al. The immunological synapse: a molecular machine controlling T cell activation. Science. 1999;285:221–227. 25888702

[40] Probst HC , McCoy K , Okazaki T , Honjo T , van den Broek M . Resting dendritic cells induce peripheral CD8+ T cell tolerance through PD‐1 and CTLA‐4. Nat Immunol. 2005;6. 10.1038/ni1165 15685176

[41] Yin Y , Wu C , Song J , et al. DNA immunization with fusion of CTLA‐4 to hepatitis B virus (HBV) core protein enhanced Th2 type responses and cleared HBV with an accelerated kinetic. PLoS ONE. 2011;6:e22524. 21799884 10.1371/journal.pone.0022524 PMC3142188

[42] Bendix I , Pfueller CF , Leuenberger T , et al. MAPK3 deficiency drives autoimmunity via DC arming. Eur J Immunol. 2010;40:1486–1495. 20186879 10.1002/eji.200939930

[43] Hu L , Chen G , Yu H , Qiu X . Clinicopathological significance of RASSF1A reduced expression and hypermethylation in hepatocellular carcinoma. Hep Intl. 2010;4:423–432. 10.1007/s12072-010-9164-8 PMC2836437 20305761

[44] Dong X , He H , Zhang W , Yu D , Wang X , Chen Y . Combination of serum RASSF1A methylation and AFP is a promising non‐invasive biomarker for HCC patient with chronic HBV infection. Diagnostic pathology. 2015;10:133. 26238200 10.1186/s13000-015-0317-x PMC4545862

[45] Csepregi A , Ebert M , Röcken C , et al. Promoter methylation of CDKN2A and lack of p16 expression characterize patients with hepatocellular carcinoma. BMC Cancer. 2010;10:317. 20569442 10.1186/1471-2407-10-317 PMC2927998

[46] Bournazos S , Ravetch JV . Fcγ Receptor Function and the Design of Vaccination Strategies. Immunity. 2017;47:224–233. 28813656 10.1016/j.immuni.2017.07.009 PMC5573140

[47] Wieland A , Shashidharamurthy R , Kamphorst AO , et al. Antibody effector functions mediated by Fcγ‐receptors are compromised during persistent viral infection. Immunity. 2015;42:367–378. 25680276 10.1016/j.immuni.2015.01.009 PMC4339104

[48] Yamada DH , Elsaesser H , Lux A , et al. Suppression of Fcγ‐receptor‐mediated antibody effector function during persistent viral infection. Immunity. 2015;42:379–390. 25680277 10.1016/j.immuni.2015.01.005 PMC4334737

[49] Martín‐Fontecha A , Thomsen LL , Brett S , et al. Induced recruitment of NK cells to lymph nodes provides IFN‐γ for T H 1 priming. Nat Immunol. 2004;5:1260. 15531883 10.1038/ni1138

[50] Sibéril S , Dutertre C‐A , Boix C , et al. Molecular aspects of human FcγR interactions with IgG: functional and therapeutic consequences. Immunol Lett. 2006;106:111–118. 16797726 10.1016/j.imlet.2006.05.009

[51] Nimmerjahn F , Ravetch JV . Fcγ receptors as regulators of immune responses. Nat Rev Immunol. 2008;8:34. 18064051 10.1038/nri2206

[52] Call ME , Wucherpfennig KW . Molecular mechanisms for the assembly of the T cell receptor–CD3 complex. Mol Immunol. 2004;40:1295–1305. 15072848 10.1016/j.molimm.2003.11.017 PMC4515969

[53] Roifman CM . CD3δ immunodeficiency. Curr Opin Allergy Clin Immunol. 2004;4:479–484. 15640687 10.1097/00130832-200412000-00002

[54] Salmond RJ , Filby A , Qureshi I , Caserta S , Zamoyska R . T‐cell receptor proximal signaling via the Src‐family kinases, Lck and Fyn, influences T‐cell activation, differentiation, and tolerance. Immunol Rev. 2009;228:9–22. 19290918 10.1111/j.1600-065X.2008.00745.x

[55] Wong WF . Inhibitors of the tyrosine kinase signaling cascade for asthma. Curr Opin Pharmacol. 2005;5:264–271. 15907913 10.1016/j.coph.2005.01.009

[56] Yang Z , Zhang J , Lu Y , et al. Aspartate aminotransferase‐lymphocyte ratio index and systemic immune‐inflammation index predict overall survival in HBV‐related hepatocellular carcinoma patients after transcatheter arterial chemoembolization. Oncotarget. 2015;6:43090–43098. 26506519 10.18632/oncotarget.5719 PMC4767493

[57] Sun H‐C , Zhang W , Qin L‐X , et al. Positive serum hepatitis B e antigen is associated with higher risk of early recurrence and poorer survival in patients after curative resection of hepatitis B‐related hepatocellular carcinoma. J Hepatol. 2007;47:684–690. 17854945 10.1016/j.jhep.2007.06.019

[58] Ferry O , Nathalie F , Marcel C , et al. Differential expression regulation of the alpha and beta subunits of the PA28 proteasome activator in mature dendritic cells. Journal of Immunology. 2005;174:7815–7822. 10.4049/jimmunol.174.12.7815 15944286

[59] Lee N , Ishitani A , Geraghty DE . HLA‐F is a surface marker on activated lymphocytes. Eur J Immunol. 2010;40:2308–2318. 20865824 10.1002/eji.201040348 PMC3867582

[60] Sadler AJ , Williams BR . Interferon‐inducible antiviral effectors. Nat Rev Immunol. 2008;8:559–568. 18575461 10.1038/nri2314 PMC2522268

[61] Liu SY , Sanchez DJ , Cheng G . New developments in the induction and antiviral effectors of type I interferon. Curr Opin Immunol. 2011;23:57–64. 21123041 10.1016/j.coi.2010.11.003 PMC3822007

[62] Cheriyath V , Leaman DW , Borden EC . Emerging Roles of FAM14 Family Members (G1P3/ISG 6–16 and ISG12/IFI27) in Innate Immunity and Cancer. J Interferon Cytokine Res. 2011;31:173–181. 20939681 10.1089/jir.2010.0105 PMC6468951

[63] Brass AL , Huang I‐C , Benita Y , et al. The IFITM Proteins Mediate Cellular Resistance to Influenza A H1N1 Virus, West Nile Virus, and Dengue Virus. Cell. 2009;139:1243–1254. 20064371 10.1016/j.cell.2009.12.017 PMC2824905

[64] Singh R , Jamieson A , Cresswell P . GILT is a critical host factor for Listeria monocytogenes infection. Nature. 2008;455:1244–1247. 18815593 10.1038/nature07344 PMC2775488

