# Supplementary figures and images for: Identification of key genes and pathways associated with different immune statuses of hepatitis B virus infection
Source: J Cell Mol Med. 2019 Sep 29;23(11):7474–89. doi: 10.1111/jcmm.14616 (PMC6815815; doi:10.1111/jcmm.14616)

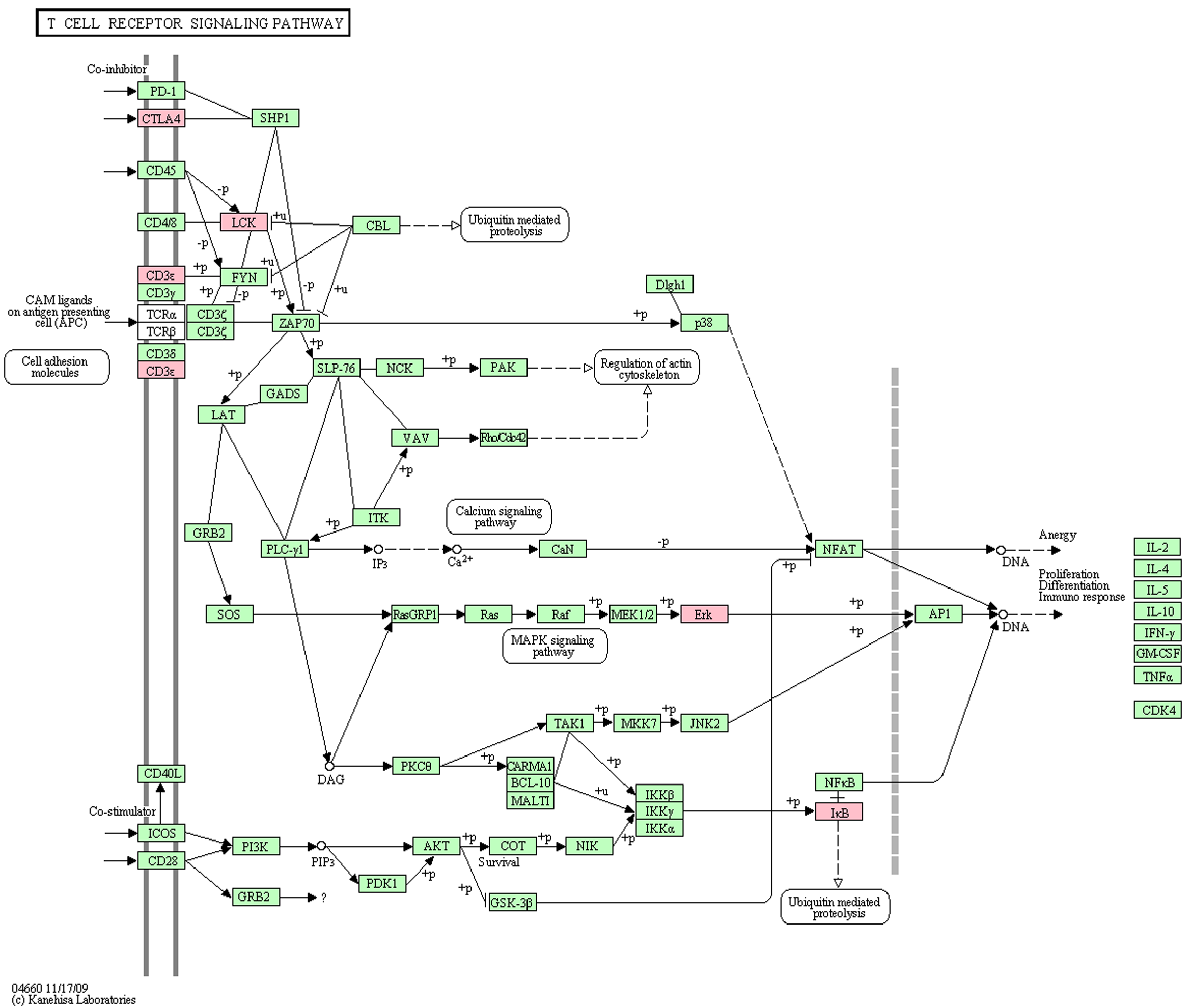

Supplement: Supplementary file 1 [file JCMM-23-7474-s001.tif]
