# Supplementary material for: Identification of key genes and pathways associated with different immune statuses of hepatitis B virus infection
Source: J Cell Mol Med. 2019 Sep 29;23(11):7474–89. doi: 10.1111/jcmm.14616 (PMC6815815; doi:10.1111/jcmm.14616)
Supplement: Supplementary file 2 [file JCMM-23-7474-s002.docx]

Supplementary Table 1 The characteristics and clinical features of patients used for developing gene expression profile of HBV infection

| Features | AH | CA1 | CA2 | CH1 | CH2 | N |
| --- | --- | --- | --- | --- | --- | --- |
| Individuals | 9 | 16 | 12 | 29 | 8 | 8 |
| Age |  |  |  |  |  |  |
| Mean±SD  Median (range) | 33.2±7.0  33(21-43) | 26.3±8.3  25(14-45) | 26.3±8.6  24(15-43) | 34.1±7.3  34(18-49) | 39.8±14.5  40(24-64) | 32.1±10.2  28(24-52) |
| Sex (%) |  |  |  |  |  |  |
| Male  Femal | 7(78)  2(22) | 6(37.5)  10(62.5) | 5(42)  7(58) | 23(79)  6(21) | 8(100)  0(0) | 5(62.5)  3(37.5) |
| HBV DNA (log10 copies/mL) |  |  |  |  |  |  |
| Mean±SD | 5.37±1.87 | 7.37±0.86 | 3.03±0.07 | 6.5±1.09 | 6.23±1.64 |  |
| ALT (U/L) |  |  |  |  |  |  |
| Median (range) | 1029(57-2645) | 20.5(10-37) | 16(10-36) | 337(73-2286) | 144(116-249) |  |
| AST (U/L) |  |  |  |  |  |  |
| Median (range) | 439(130-1374) | 21(10-30) | 13(10-32) | 194(55-1555) | 102.5(58-204) |  |

AH: Acute hepatitis B; CA1: Immune-tolerance phase; CA2: Inactive phase; CH1: Immune-clearance (E antigen positive) phase; CH2: Immune-clearance (E antigen negative) phase; N: healthy control; ALT: Alanine aminotransferase; AST: Aspartate aminotransferase. The normal value of clinical indicators was as follows: ALT, ≤40 IU/L; AST, ≤40 IU/L; HBV DNA≤500IU/ml.

Supplementary Table 2 The characteristics of patients and healthy controls used for analysis of the expression of FcγRs in different immune status of HBV infection

| Features | HBISC | CA | CH | N |
| --- | --- | --- | --- | --- |
| Individuals | 12 | 20 | 32 | 14 |
| Age |  |  |  |  |
| Mean±SD | 47.17±11.5 | 28.20±5.7 | 33.22±10.4 | 28.86±4.1 |
| Median (range) | 48 (26-68) | 28 (14-37) | 33 (17-63) | 28 (24-40) |
| Gender, number (%) | | | | |
| Male | 9 (75) | 7 (35) | 24 (75) | 8 (57.1) |
| Female | 3 (25) | 13 (65) | 8 (25) | 6 (42.9) |
| HBV DNA（log10copies/ml） | 5.76±1.47 | 8.26±0.26 | 7.34±1.40 |  |
| ALT (U/L)  Median (range） | 1170 (550-3693) | 28 (2-49) | 187 (57-1900) |  |
| AST (U/L)  Median (range） | 914 (224-2785) | 24.5 (15-37) | 109.5 (61-1060) |  |
| HBsAg  Median (range） | 814.6 (175-5479) | 53692 (8773-85613) | 4781 (56.48-124925) |  |

Normal values: ALT≤40U/L; AST≤40U/L; HBV≤500IU/ml. HBISC: Hepatitis B successfully cleared; CA: Immune-tolerance phase; CH: Immune-clearance phase; N: Healthy controls; HBsAg: Hepatitis B surface antigen; AST: Aspartate transaminase; ALT: Alanine aminotransferase.

Supplementary Table 3 The characteristics of patients and healthy controls used for analysis of differential expression of FcγRs on immune cells

| Features | HBISC | CA | CH | N |
| --- | --- | --- | --- | --- |
| Individuals | 7 | 16 | 31 | 20 |
| Age | 39.86±11.70 | 30.69±4.98 | 33.68±9.88 | 28.35±7.20 |
| Gender, number (%) |  |  |  |  |
| Male | 1 (14.3 | 7 (43.8) | 25 (80.6) | 14 (0.7) |
| Female | 6 (85.7) | 9 (56.2) | 6 (19.4) | 6 (0.3) |
| HBV DNA  (log_10_ copies/mL) | 4.83±1.96 | 8.33±0.18 | 6.58±1.94 | — |
| HBsAg  (log_10_ IU/mL) | 2.21±1.30 | 4.78±0.21 | 3.56±0.90 | — |
| ALT(U/L)  Median (range） | 599.2 (110.3-1685.6) | 38.5 (23.0-45.0) | 588.8 (172.5-2248) | 24.5 (3.0-49.0) |
| AST(U/L)  Median (range） | 160.4 (59.9-1352.8) | 30.5  (11.0-46.0) | 245.6  (64.3-1384.0) | 21.5  (13.0-36.0) |

Normal values: ALT≤40U/L; AST≤40U/L; HBV≤500IU/ml. HBISC: Hepatitis B successfully cleared; CA: Immune-tolerance phase; CH: Immune-clearance phase; N: Healthy controls; HBsAg: Hepatitis B surface antigen; AST: Aspartate transaminase; ALT: Alanine aminotransferase.

Supplementary Table 4 The percent of FcγRs in peripheral blood’s lymphocytes of patients with HBC infection

| Groups | N | HBISC | CH | CA |
| --- | --- | --- | --- | --- |
| A: Total NK cell and its subsets | | | | |
| Total NK cells  (%) | 18.69  (13.55,23.73) | 6.67  (5.72,9.16) | 7.80  (5.41,11.56) | 15.13  (9.81,18.80） |
| CD3^-^CD56^+^  (%) | 18.31  (12.22,22.97) | 6.26  (5.28,7.98) | 7.16  (4.57,10.10) | 13.74  (9.34,17.22) |
| CD3^-^ CD16^+^  (%) | 17.99  (12.99,22.80) | 6.07  (5.12,8.59) | 7.11  (4.23,10.12) | 14.49  (8.62,18.14) |
| CD3^-^CD56^+^CD16^-^（%） | 0.73  (0.59,0.99) | 0.60  (0.57,0.63) | 1.04  (0.52,1.45) | 0.71  (0.56,0.83) |
| CD3^-^CD56^+^CD16^+^（%） | 17.62  (11.69,22.29) | 5.66  (4.71,7.42) | 6.00  (3.61,9.13) | 13.22  (7.94,16.57) |
| CD3^-^CD56^-^CD16^+^(%) | 0.65  (0.44,0.87) | 0.45  (0.41,1.18） | 0.71  (0.45,0.89) | 1.05  (0.55,1.44) |
| B: Subsets of B cells | | | | |
| CD3^-^CD19^+^ (%) | 7.72  (6.31,11.20) | 6.75  (5.75,9.92) | 12.40  (8.12,17.20) | 7.91  (6.73,9.95) |
| CD3^-^CD5^+^ (%) | 2.22  (1.51,3.54) | 1.82  (1.55,3.22) | 3.95  (2.49,5.93) | 2.73  (1.80,4.35) |
| CD3^-^CD19^+^CD5^-^ (%) | 5.95  (4.27，7.05) | 5.78  (3.40,6.95) | 8.10  (6.18,12.21) | 5.34  (4.66,6.99) |
| CD3^-^CD19^+^CD5^+^  (%) | 1.45  (1.14,2.71) | 1.57  (0.87,2.93) | 3.35  (1.63,5.49) | 1.85  (1.20,3.27) |
| CD3^-^CD19^+^CD32^+^ (%) | 7.09  (5.42,9.84) | 6.64  (4.99,9.09) | 11.68  (7.84,16.49) | 7.28  (6.06，9.49) |
| CD3-CD5+CD32+ (%) | 1.46  (1.26,2.64) | 1.7  1(0.97,2.63) | 3.37  (1.94,5.18) | 1.98  (1.26,2.52) |
| CD3^-^CD19^+^ CD5^-^CD32^+^ (%) | 5.81  (4.18,6.85) | 5.47  (3.22,6.13) | 7.77  (5.82,10.59) | 5.22  (4.41,6.68) |
| CD3^-^CD19^+^ CD5^+^ CD32^+^ (%) | 1.34  (1.04,2.57) | 1.49  (0.73,2.70) | 3.26  (1.58,5.09) | 1.58  (1.12,3.17) |
| C: Subsets of monocytes | | | | |
| CD14^high^CD16^+^ | 3.59  (2.89,5.49) | 7.96  (4.64,12.00) | 8.04  (5.82,10.80) | 4.84  (2.46,6.71) |
| CD14^+^CD32^+^ | 24.08  (2.11.86.83) | 3.39  (1.79,76.00) | 79.4  (3.18,87.00) | 73.65  (2.16,84.93) |
| CD14+CD64+ | 84.15  (75.40.88.88) | 83.70  (77.10,90.40) | 86.60  (79.70,90.20) | 83.75  (79.80,88.25) |

HBISC: Hepatitis B successfully cleared; CA: Immune-tolerance phase; CH: Immune-clearance phase; N: healthy controls
